# Optimizing respiratory virus replication in human bronchial/tracheal epithelial air–liquid interface cultures through practical quality indicators and key characteristics

**DOI:** 10.1099/jgv.0.002361

**Published:** 2026-11-05

**Authors:** Satoko Sugimoto, Nozomi Shiwa-Sudo, Miyuki Kawase, Yohei Kume, Hisao Okabe, Shun Hiruta, Jun Shirota, Sakurako Norito, Hayato Go, Koichi Hashimoto, Ken-Ichi Hanaki, Noriyo Nagata, Kazuya Shirato

**Affiliations:** 1 Department of Respiratory Viruses, National Institute of Infectious Diseases, Japan Institute for Health Security, Tokyo, Japan; 2 Research Center for Biosafety, Laboratory Animal and Pathogen Bank, National Institute of Infectious Diseases, Japan Institute for Health Security, Tokyo, Japan; 3 Department of Infectious Disease Pathology, National Institute of Infectious Diseases, Japan Institute for Health Security, Tokyo, Japan; 4 Department of Pediatrics, School of Medicine, Fukushima Medical University, Fukushima, Japan

**Keywords:** air–liquid interface, human bronchial/tracheal epithelial cell air–liquid interface (HBTEC-ALI) culture, KRT5, respiratory virus, transepithelial electrical resistance, viral replication

## Abstract

The human bronchial/tracheal epithelial air–liquid interface (HBTEC-ALI) culture system is widely used for isolating and propagating respiratory viruses; however, variability in differentiation status complicates quality control and may influence viral replication efficiency. Practical criteria for selecting optimally differentiated cultures remain limited. In this study, we classified HBTEC-ALI cultures into three morphological categories – Thick, Intermediate and Thin – based on cell size, ciliary activity and mucus production. These categories were associated with epithelial thickness and expression of the basal cell marker *KRT5*. Thin-type cultures exhibited significantly reduced transepithelial electrical resistance and diminished apical glycan staining, suggesting compromised barrier integrity and altered apical glycan composition. We next evaluated replication of multiple respiratory viruses. Most viruses tended to replicate more efficiently in Thick- and Intermediate-type cultures, whereas adenovirus replication was enhanced in Thin-type cultures. Bocavirus replication did not differ between types. Our findings suggest that morphological assessment of HBTEC-ALI cultures is associated with functional barrier status and virus-specific replication efficiency. In addition, the association between *KRT5* expression and morphological phenotypes suggests that *KRT5* expression may reflect differences in epithelial characteristics among HBTEC-ALI cultures. These results may provide a practical framework for selecting HBTEC-ALI cultures and may help improve the reproducibility and efficiency of respiratory virus isolation and propagation.

**Impact Statement**

Reliable isolation of respiratory viruses is essential for surveillance and diagnostics. Although the human bronchial/tracheal epithelial cell air–liquid interface (HBTEC-ALI) culture system closely mimics the human airway and is widely used for virus propagation, controlling the degree of epithelial differentiation remains technically challenging and batch-to-batch variability is difficult to avoid. Because strict control of epithelial differentiation is technically challenging, selecting cultures with appropriate morphological features represents a practical alternative. We show that easily recognizable morphological characteristics reflect functional differences that influence viral replication and that optimal culture conditions vary depending on the virus. By linking readily observable culture morphology with quantitative characteristics, particularly *KRT5* expression, this work identifies *KRT5* as a potentially useful indicator of HBTEC-ALI culture status and provides a framework for improving the reproducibility of respiratory virus isolation and propagation.

## Introduction

The human bronchial/tracheal epithelial cell air–liquid interface (HBTEC-ALI) culture system is an extremely useful platform for isolating and propagating a broad range of respiratory viruses [1]. Notably, it supports viruses that are difficult to replicate in conventional immortalized cell lines, such as bocavirus, human coronavirus (HCoV)-HKU1 and rhinovirus [2–4]. HBTEC-ALI cultures provide a differentiated airway epithelium that closely mimics the *in vivo* environment of the human respiratory tract, making them particularly valuable for virological studies.

Both the epithelial thickness and transepithelial electrical resistance (TEER) of HBTEC-ALI cultures are known to be influenced by the passage number of the primary epithelial cells used [5–8]. However, even when cultured using identical cell stocks and culture procedure, the differentiation status of HBTEC-ALI cultures can vary across batches and among individual wells. This variability complicates quality control and may influence viral replication efficiency. However, the culture characteristics associated with optimal virus growth in this system have not been well defined. Therefore, researchers must empirically evaluate and select HBTEC-ALI cultures that are appropriate for respiratory virus studies.

The degree of airway epithelial differentiation is closely associated with the composition of distinct epithelial cell types. A fully differentiated epithelium is characterized by a pseudostratified layer of ciliated, basal and secretory cells (including club and goblet cells). *In vitro* airway epithelium models are often assessed using well-established differentiation markers to determine cell-type composition and maturation. Basal cell markers (e.g. *KRT5*), secretory markers (e.g. *SCGB1A1*), goblet cell markers (e.g. *MUC5AC*) and ciliated markers (e.g. *FOXJ1* and acetylated *α*-tubulin) are commonly used to quantify differentiation status, and their expression has been validated in both two-dimensional and three-dimensional culture systems [9–11].

A functional epithelial barrier is a key indicator of proper airway epithelial differentiation. Epithelial cells are interconnected by tight junctions, which maintain epithelial integrity and regulate paracellular permeability [7, 12]. On the apical surface, the mucus layer and the periciliary layer (PCL) [13], along with the glycocalyx, play crucial roles in mucociliary clearance and defence against inhaled pathogens [14]. Sialic acids on the apical surface serve as receptors for many respiratory viruses [15], and keratan sulphate contributes to PCL structure and barrier function [14].

In this study, we used readily observable morphological variation in HBTEC-ALI cultures as a starting point to examine its relationship with quantitative structural and functional characteristics relevant to respiratory virus infection. Our objective was to identify practical indicators that reflect HBTEC-ALI culture status and may facilitate the selection of appropriately differentiated cultures for virological applications.

## Methods

### Viruses

Passage 0 (P0) clinical isolates, initially isolated using HBTEC-ALI cultures, were propagated once on HBTEC-ALI cultures, and the resulting P1 viral stocks were used in the present study. The P0 sequences are listed below: HCoV-HKU1, Fukushima_H815_2020 (GenBank accession no. LC654447) [16]; HCoV-OC43, Fukushima_O502_2019 (LC687400) [17]; HCoV-NL63, Fukushima_H552_2019 (LC687396) [17]; HCoV-229E, Fukushima_H829_2020 (LC654445) [18]; human parainfluenza virus (HPIV) 1, PIV1_Fukushima_O77_2018 (LC764863) [17]; HPIV2, PIV2_Fukushima_O13_2018 (LC720864) [17]; HPIV3, PIV3_Fukushima_O695_2019 (LC720878) [17]; HPIV4a, PIV4a_Fukushima_H725_2019 (LC706553) [19]; HPIV4b, PIV4b_Fukushima_OR476_2022 (LC706556) [19]; human rhinovirus (HRV) A, HRV_A16_Fukushima_H561_2019 (LC699418) [20]; HRVB, HRV_B6_Fukushima_OR636_2022 (LC769226) [17]; HRVC, HRV_C3_Fukushima_H399_2018 (LC699425) [20]; influenza C virus (FluC), C/Fukushima/O181/2018 (LC720285-LC720291) [17]; respiratory syncytial virus (RSV), RSVB_Fukushima_HR128_2021 (LC769227) [21]; human bocavirus 1 (HBoV1), Fukushima_H181_2018 (LC651167) [22]; adenovirus (ADV) 2 (species C), ADV2_Fukushima_O170_2018 (LC703522) [17]; and ADV3 (species B), ADVB3_Fukushima_H739_2019 (LC703523) [17].

### Cells

Primary human bronchial/tracheal epithelial (HBTE) cells (FC-0035; lot nos. 01335, 06288 and 09439; Lifeline Cell Technology, Frederick, MD, USA) were purchased. HBTEC-ALI cultures were established as described below. Expansion of HBTE cells in submerged culture was performed with a 1 : 1 mixture of PneumaCult-Ex Medium (05008; STEMCELL Technologies, Vancouver, Canada) and PneumaCult-Ex Plus Medium (05040; STEMCELL Technologies) supplemented with hydrocortisone (07904; STEMCELL Technologies). Expanded HBTE cells were removed using EDTA–trypsin solution (0.02% EDTA, 0.1% trypsin; Cosmo Bio, Tokyo, Japan) and trypsin inhibitor (T6522; Sigma, St. Louis, MO, USA). A total of 1×10^5^ cells per insert were seeded into the collagen-coated (Cellmatrix Type IV; Nitta Gelatin, Osaka, Japan) apical chamber of a Costar 6.5 mm Transwell with a 0.4 µm pore polyester membrane insert (3470; Corning, Kennebunk, ME, USA). After 1 to 2 days of culture, HBTE cells were generated by ALI culture using PneumaCult-ALI Medium (ST-05001; STEMCELL Technologies) supplemented with heparin solution (07980; STEMCELL Technologies) and hydrocortisone. After at least 3 weeks, differentiated cells were independently evaluated by two experienced investigators using an inverted microscope. Classification was based on the overall morphological appearance of the cultures, including cell size, ciliary activity and mucus production, and cultures were categorized into three morphological types. Cells were cultured in a 5% CO_2_ incubator at 37 °C before virus inoculation.

### Histology and immunohistochemistry

Differentiated HBTEC-ALI cultures were fixed in 4% paraformaldehyde, embedded in paraffin, sectioned and stained with haematoxylin and eosin. Images were captured with a BZ-X810 fluorescence microscope (Keyence, Osaka, Japan). Epithelial thickness in haematoxylin and eosin–stained images was measured using a BZ-X800 Analyzer (Keyence) at three locations per image in three wells for each group. For immunohistochemical analysis, a polymer-based detection system (Nichirei-Histofine Simple Stain MAX PO; Nichirei Biosciences, Tokyo, Japan) was used. Paraffin sections were heated at 65 °C for 1 h and then deparaffinized. Antigen retrieval was carried out using Deparaffinization/Antigen Retrieval Solution pH6 (Nichirei Biosciences) in a pressure cooker at 95 °C for 20 min. Endogenous peroxidase activity was blocked by treating the sections with 0.3% hydrogen peroxide in methanol at room temperature for 30 min. Non-specific binding was blocked with 5% normal goat serum (005-000-121; Jackson ImmunoResearch Laboratories, Inc.) for 30 min at room temperature. The sections were then incubated overnight at 4 °C with primary antibodies diluted in PBS containing 1% BSA and 0.1% sodium azide. The antibodies were diluted as follows: TUBA (clone 6-11B-1, sc-23950, 1 : 4000; Santa Cruz Biotechnology, Dallas, TX, USA), CC10 (clone E-11, sc-365992, 1 : 500; Santa Cruz), cytokeratin 5 (CK5) (clone E2T4B, 71536, 1 : 500; Cell Signalling Technology, Danvers, MA, USA) and MUC5AC (clone 45M1, Ab3649, 1 : 100; Abcam, Cambridge, UK). The following day, the sections were incubated with Histofine Simple Stain MAX PO (M) or (R) reagent (Nichirei Biosciences) for 30 min at room temperature. Peroxidase activity was detected with 3,3′-diaminobenzidine (Sigma), and counterstaining was performed with haematoxylin. Multiplex staining was performed using an automated immunohistochemistry system (Bond RXm; Leica Biosystems, Nussloch, Germany). Primary antibodies were diluted in 1×Antibody Diluent/Block (ARD1001EA; Akoya Biosciences, Marlborough, MA, USA) at the same dilution factors as described above. All steps were performed in accordance with the manufacturer’s protocol. Paraffin sections were heated at 65 °C for 1 h. Deparaffinization was performed using Bond Dewax Solution (AR9222; Leica Biosystems), followed by alcohol and Bond Wash solution (AR9590; Leica Biosystems). Antigen retrieval was performed using Bond Epitope Retrieval Solution 1 (AR9961; Leica Biosystems) for 20 min at 100 °C. Blocking was performed with peroxide block reagent for 5 min at ambient temperature. The primary antibody reaction was performed for 30 min at ambient temperature. Colour development was performed with Opal 520, Opal 570, Opal 620 and Opal 690 (Akoya Biosciences) for 10 min at ambient temperature. Nuclei were stained for 5 min at ambient temperature. Images were captured using a PhenoImager Fusion (Akoya Biosciences) at high magnification (×200). The acquired images were processed using software provided by the manufacturer (Instrument software HT 2.0 Phenochart 2.0 and inForm 3.0; Akoya Biosciences).

### Real-time PCR for identification of cell markers

Total RNA was extracted from cells in one well using ISOGEN (NIPPON GENE, Tokyo, Japan). cDNA was synthesized using ReverTra Ace qPCR RT Master Mix with gDNA Remover (Toyobo, Osaka, Japan) with random primers (11034731001; Roche, Basel, Switzerland). Real-time PCR was performed using THUNDERBIRD Next SYBR qPCR Mix (Toyobo) on a LightCycler 480 II instrument (Roche) under the following conditions: 95 °C for 30 s, followed by 45 cycles of 95 °C for 5 s and 60 °C for 30 s with fluorescence acquisition. A melting curve was subsequently acquired to confirm amplification specificity. The final concentration of primers was 300 nM. Relative quantification of gene expression was performed using the 2^−∆∆Ct^ method, with GAPDH as the internal control for normalization. The pre-differentiation cells were used as the control condition. Primers for amplification of the genes were obtained from previously published literature [12].

#### TEER measurements

TEER measurements were conducted using a Millicell ERS-2 device (Millipore, Burlington, MA, USA). Prior to measurement, the medium in the basolateral chamber was replaced with 800 µl of PBS, and 200 µl of PBS was added to the apical chamber. Inserts without cells were prepared as blanks. Measurements were performed in the order of two blanks followed by the samples. This procedure was repeated three times, and the average of the three measurements was used to calculate the electrical resistance of each sample. The resistance (Ω) of each sample was determined by subtracting the mean of the two blanks from its measurement. TEER (Ω⋅cm^2^) was obtained by multiplying the resistance by the effective surface area of the membrane (0.33 cm^2^).

### Lectin and keratan sulphate staining

Cultures were fixed with 4% paraformaldehyde for 30 min at room temperature with 300 µl on the apical side and 500 µl on the basolateral side. For staining, lectins and antibodies were diluted in PBS containing 2% BSA. FITC-conjugated *Sambucus nigra* lectin (SNA; L32479, 5 µg ml^−1^; Invitrogen, Waltham, MA, USA), Alexa Fluor 594-conjugated peanut agglutinin (PNA; L32459, 5 µg ml^−1^; Invitrogen) or Anti-Keratan Sulphate (R-10G) (RIT-M001, 5 µg ml^−1^; Cosmo Bio) was added to the apical side at 50 µl per well and incubated for 1 h at room temperature. For detection of the keratan sulphate antibody, Alexa Fluor 488-conjugated goat anti-mouse IgG (H+L) secondary antibody (A28175, 1 : 200; Invitrogen) was added to the apical side at 50 µl per well and incubated for 1 h at room temperature. Fluorescence signals were observed using a BZ-X810 fluorescence microscope (Keyence).

### Apical washes from Thick-type cultures

A total of 200 µl of PBS was applied to the apical surface and incubated for 10 min at 37 °C. The apical wash was then recovered by pipetting and used immediately. Protein concentration was determined to be 541 ng µl^−1^ by measuring absorbance at 280 nm using a NanoDrop One (Thermo Fisher Scientific, Waltham, MA, USA). The apical wash was subsequently diluted 1 : 1 with Dulbecco’s modified Eagle’s medium (DMEM)-high glucose (D5796; Sigma) containing 2% FCS (175012; Nichirei Biosciences, Tokyo, Japan). The virus was diluted with this diluted apical wash at a ratio of 1 : 4, resulting in a final apical wash concentration equivalent to 2.5-fold dilution of the original apical wash.

### Viral inoculation in HBTEC-ALI cultures

The P1 viral stocks were diluted in DMEM supplemented with 1% FCS to achieve the following viral genome copy numbers per 50 µl inoculation volume: 10^3^ copies for HCoV-OC43; 10^5^ copies for HCoV-HKU1, HCoV-229E, HPIV1–4, HRVA, HRVB, HRVC, FluC and HBoV1; 10^6^ copies for HCoV-NL63, ADVC and ADVB; and 10^9^ copies for RSV. The diluted viruses were then inoculated onto HBTEC-ALI cultures and incubated at 34 °C in 5% CO_2_. After overnight incubation, the apical surface of the HBTEC-ALI cultures was washed four times with DMEM containing 1% FCS. The wash fluid from the fourth washing step was collected and stored at −80 °C. The ALI culture was maintained thereafter, and the medium in the basolateral chamber was replaced every 3 to 4 days. To collect replicated virus, the apical surface was washed at 4, 7, 14 and 21 days post infection (dpi), and a total of 400 µl of wash fluid (200 µl×2 washes) was collected at each time point and stored at −80 °C.

### Detection of respiratory viruses

Nucleic acids were extracted from 100 µl of wash fluid using QIAamp 96 Virus QIAcube HT kit (Qiagen, Hilden, Germany), in accordance with the manufacturer’s instructions, except that the elution step was performed by centrifugation. For detection of RSV, RNA was directly used to prepare the reaction mixture with AgPath-ID One-Step RT-PCR Reagent (Thermo Fisher Scientific). Real time RT-PCR was performed on a LightCycler 480 II instrument (Roche) using the following conditions: reverse transcription was performed at 50 °C for 10 min, followed by 95 °C for 10 min, and then 45 cycles of 95 °C for 15 s, 56 °C for 30 s with fluorescence acquisition and 72 °C for 15 s, followed by a cooling step at 40 °C for 30 s. For other viruses, reverse transcription was carried out using ReverTra Ace (Toyobo) with a combination of random primers (Roche) and oligo-dT primers (Qiagen). The synthesized cDNA was then used to prepare the reaction mixture with LightCycler 480 Probes Master (Roche). The cycling conditions for real time PCR were 95 °C for 10 min, followed by 45 cycles of 95 °C for 10 s and 60 °C for 30 s with fluorescence acquisition. Primer and probe sequences for HCoV-229E were as follows: forward primer, GAGTCAGGCAACACTGTGGTC; reverse primer, TGTTGTTGCATTTCTCTAGTGAATG; and probe, AGAGTGACTGTGCCCAAAGACCATCCAC [23]. Sequences for the other viruses have been described previously [1]. The final concentrations of primers and probes used for RSV were as follows: primers, 200 nM; probe, 50 nM. For the other viruses, the final concentrations were as follows: primers, 900 nM, and probe, 200 nM.

### Statistics

Statistical analysis was performed using GraphPad Prism 8 (version 8.4.3; GraphPad Software, La Jolla, CA, USA). One-way ANOVA followed by Tukey’s multiple comparisons test was used for epithelial thickness measurements, mRNA expression, TEER measurements and adenovirus replication with or without apical washes. Two-way ANOVA followed by Tukey’s multiple comparisons test was used for marker protein–positive cell count data. Differences were considered statistically significant at *P*<0.05.

## Results

### Classification of HBTEC-ALI cultures

HBTEC-ALI cultures were established using the same protocol and independently evaluated by two experienced researchers. Based on cell size, ciliary motion and mucus production, cultures were classified into three morphological categories (Fig. 1a). These morphological features were closely associated with tissue thickness, as shown in Fig. 1(b, c). Cultures with dense cell distribution, exhibiting ciliary motion and mucus production throughout the well, were categorized as Thick-type ALI. In contrast, cultures with larger cells and localized ciliary motion and mucus production were categorized as Thin-type ALI. An Intermediate-type ALI category was defined for cultures with moderate cell density and partial ciliary motion and mucus production.

**Fig. 1.**
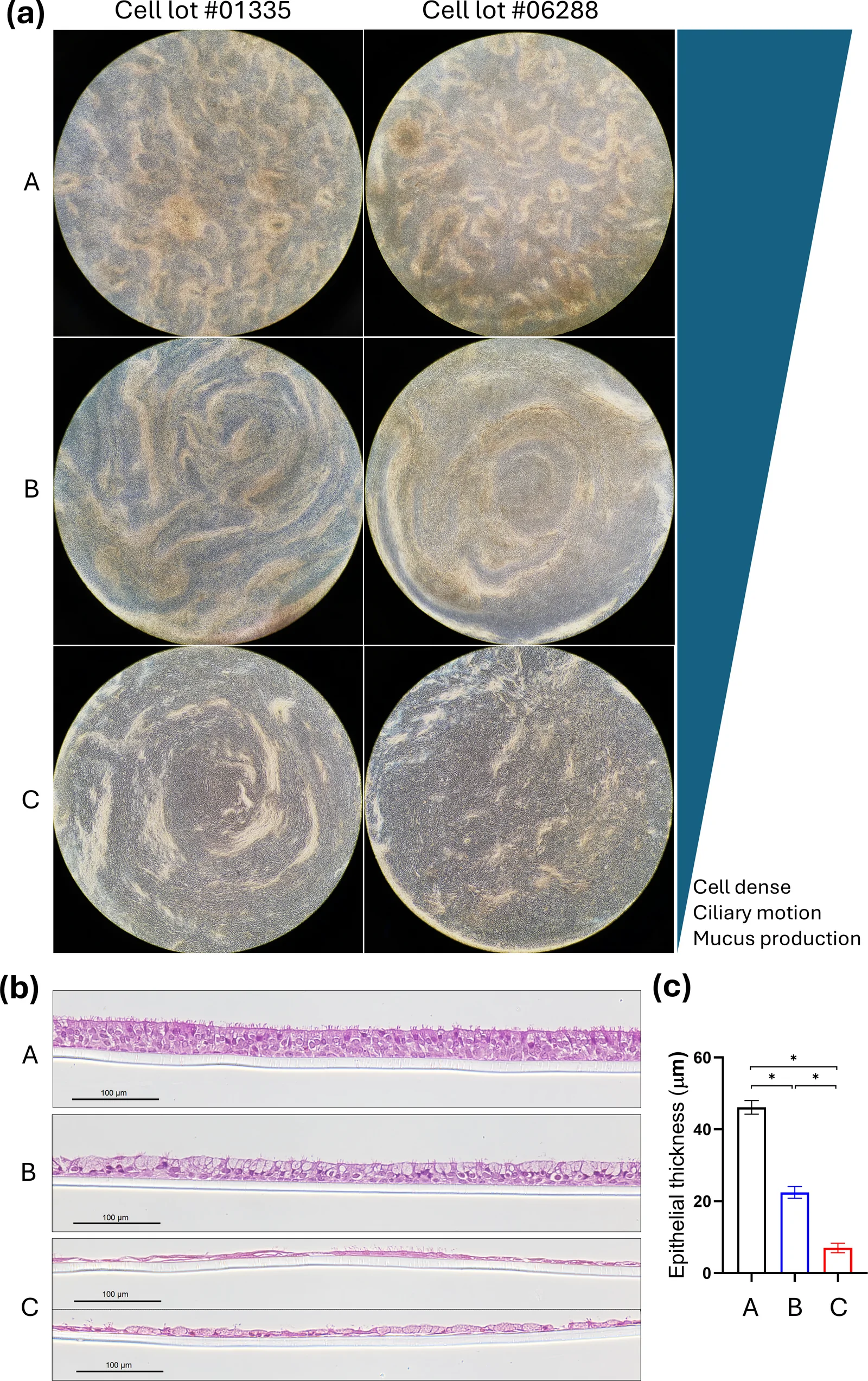
Microscopic and histological images of the classified HBTEC-ALI culture system. (**a**) Representative low-magnification images of nearly the entire well captured using a CMOS camera under an inverted microscope (40×). Thick-type ALI (**A**) shows a dense cell distribution with widespread ciliary motion and mucus production. Intermediate-type ALI (**B**) shows moderate cell density with partial ciliary activity and mucus production. Thin-type ALI (**C**) shows larger cells with localized ciliary motion and mucus production. (**b**) Representative haematoxylin and eosin–stained images of vertically sectioned, paraffin-embedded cultured tissues. Scale bar, 100 µm. (**c**) Epithelial thickness in haematoxylin and eosin–stained images was measured at three locations per image in three wells for each group. HBTEC-ALI cultures were derived from two donor lots (lot nos. 01335 and 06288), with all cultures at the same passage number. Data are presented as mean±sd. One-way ANOVA followed by Tukey’s multiple comparisons test was performed (*P*<0.05).

### Expression of respiratory epithelial cell markers in different ALI types

mRNA expression and immunostaining detected the presence of markers characteristic of ciliated, basal, secretory and goblet cells in all culture types (Fig. 2a–c). Ciliated cell markers (*TUBA1A*, *CDHR3* and *FOXJ1*) showed no significant differences among the three culture types. The basal cell marker *KRT5* exhibited the highest expression in Thick-type ALI cultures, with progressively lower levels in Intermediate- and Thin-type cultures, all showing statistically significant differences. However, the expression of other basal markers, *NGFR* and *ITGA6*, did not differ significantly. For secretory cells, the levels of *CEACAM6* and *SCGB1A1* were significantly lower in Thick-type ALI than in Thin-type ALI, while *MUC5B* expression showed no significant differences across the culture types. Regarding goblet cells, the level of *SPDEF* mRNA was significantly higher in Thick- and Intermediate-type ALI cultures than in Thin-type ALI, whereas *MUC5AC* mRNA showed no significant differences. *TSPAN8* expression was significantly higher in Intermediate-type than in Thin-type ALI but did not follow the same pattern as the culture thickness. Immunohistochemistry detected marker expression characteristic of ciliated, basal, secretory and goblet cells (Fig. 2b) in all culture types. Consistent with the mRNA data, the number of CK5-positive cells varied among culture types, with the highest numbers in Thick-type ALI and progressively lower ones in Intermediate- and Thin-type ALI (Fig. 2c). In contrast, although *CEACAM6* and *SCGB1A1* expression was higher in Thin-type ALI cultures at the mRNA level, immunostaining did not show an increased number of secretory cells in Thin-type cultures (Fig. 2a, c). The discrepancy between secretory cell-associated mRNA expression and the number of marker-positive cells may reflect differences between gene expression levels measured in bulk RNA and cell abundance assessed by immunostaining. Thus, increased expression of *CEACAM6* or *SCGB1A1* does not necessarily indicate an increased number of secretory cells. Cells with double positivity for Club cell 10 kDa protein (CC10) and mucin 5AC (MUC5AC) were observed (Fig. S1, available in the online Supplementary Material), which is consistent with normal airway epithelium [24]. Although epithelial thickness and cellular composition differ between upper and lower regions [25, 26], histological observations suggested that the morphological variations observed among cultures were associated with epithelial thickness rather than apparent loss of major epithelial cell types.

**Fig. 2.**
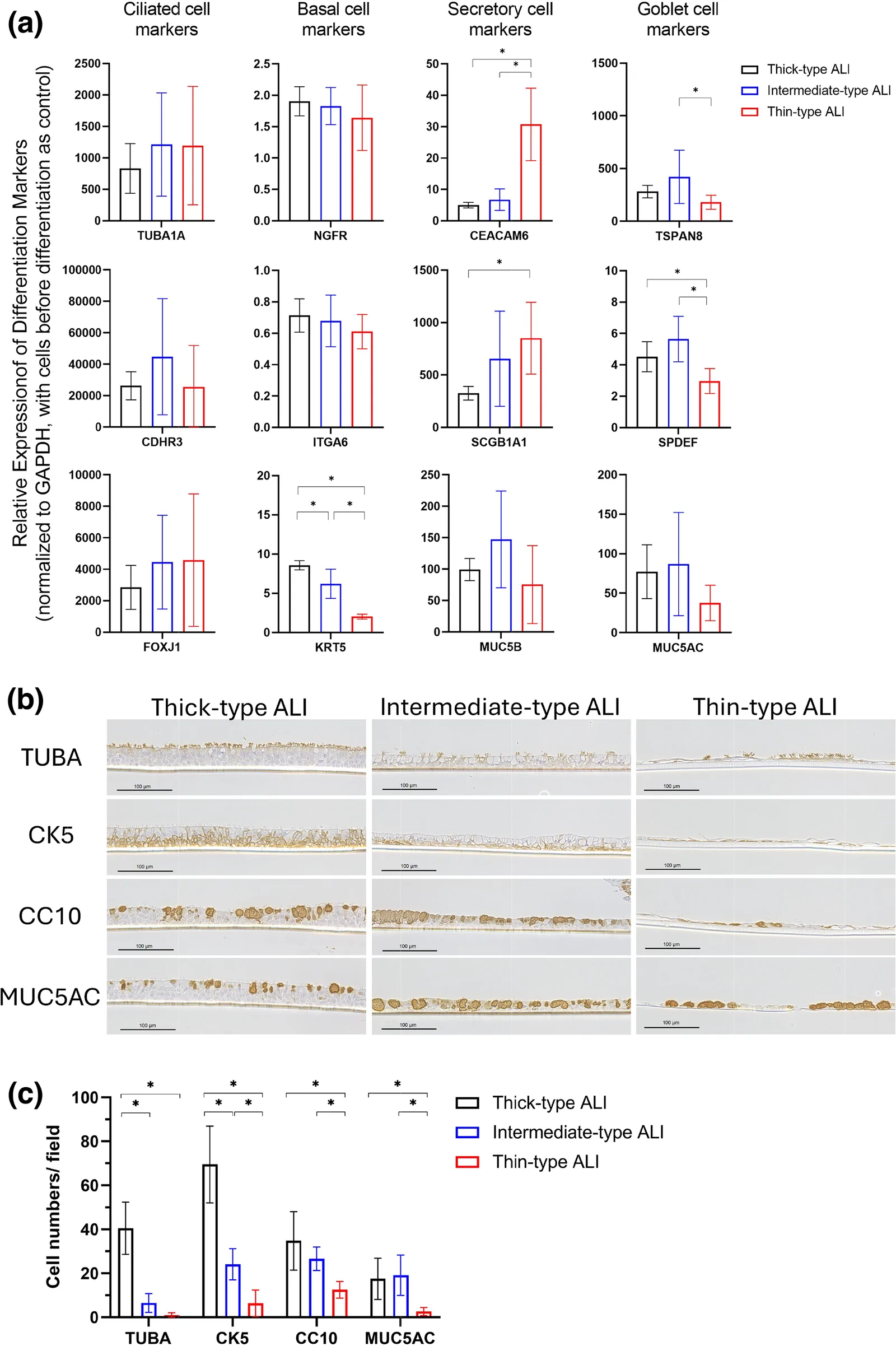
Cell differentiation marker expression in the classified HBTEC-ALI culture system. (**a**) mRNA expression of cell differentiation markers was analysed by relative quantitative RT-PCR using GAPDH as the internal control for normalization. Pre-differentiation cells were used as the control condition (*n*=4), and differentiated groups are shown with *n*=8. Data are presented as mean±sd. One-way ANOVA followed by Tukey’s multiple comparisons test was performed (*P*<0.05). Ciliary cell markers: *TUBA1A* (tubulin *α* 1A), *CDHR3* (cadherin-related family member 3), *FOXJ1* (forkhead box J1); basal cell markers: *NGFR* (nerve growth factor receptor), *ITGA6* (integrin subunit *α* 6), *KRT5*; secretory cell markers: *CEACAM6* (CEA cell-adhesion molecule 6), *SCGB1A1* (secretoglobulin family 1A member 1), *MUC5B* (mucin 5B, oligomeric mucus/gel-forming); goblet cell markers: *TSPAN8* (tetraspanin-8), *SPDEF* (SAM pointed domain–containing ETS transcription factor), *MUC5AC* (mucin 5AC, oligomeric mucus/gel-forming). (**b**) Representative immunohistochemistry images of vertically sectioned, paraffin-embedded cultured tissues using DAB staining. Differentiation markers were evaluated: TUBA (acetylated alpha tubulin) for ciliated cells, CK5 for basal cells, CC10 (club-cell-specific 10 kDa protein) for secretory cells and MUC5AC (mucin 5AC) for goblet cells. Scale bar, 100 µm. (**c**) Quantification of cell types by fluorescent quadruple staining. Four distinct cell markers were detected, and positive cells per field were automatically counted using inForm 3.0 software. Data are presented as mean±sd. Analysis was performed on eight fields per group. Statistical analysis was performed using two-way ANOVA followed by Tukey’s multiple comparisons test (*P*<0.05).

### Evaluation of epithelial barrier function and glycan integrity

TEER was used to measure the barrier function of the epithelial cell layer. TEER measurements demonstrated significantly lower resistance in Thin-type ALI cultures than in both Thick- and Intermediate-type ones (Fig. 3a). This indicates potential impairment of the barrier function in Thin-type ALI. Staining for sialic acid and keratan sulphate, important components of the glycans in the mucus layer and the PCL on the apical surface, was subsequently performed. Compared with the findings for both Thick- and Intermediate-type ALI, Thin-type ALI exhibited reduced staining intensity (Figs 3b and S2), suggesting possible alteration in the composition or integrity of these layers.

**Fig. 3.**
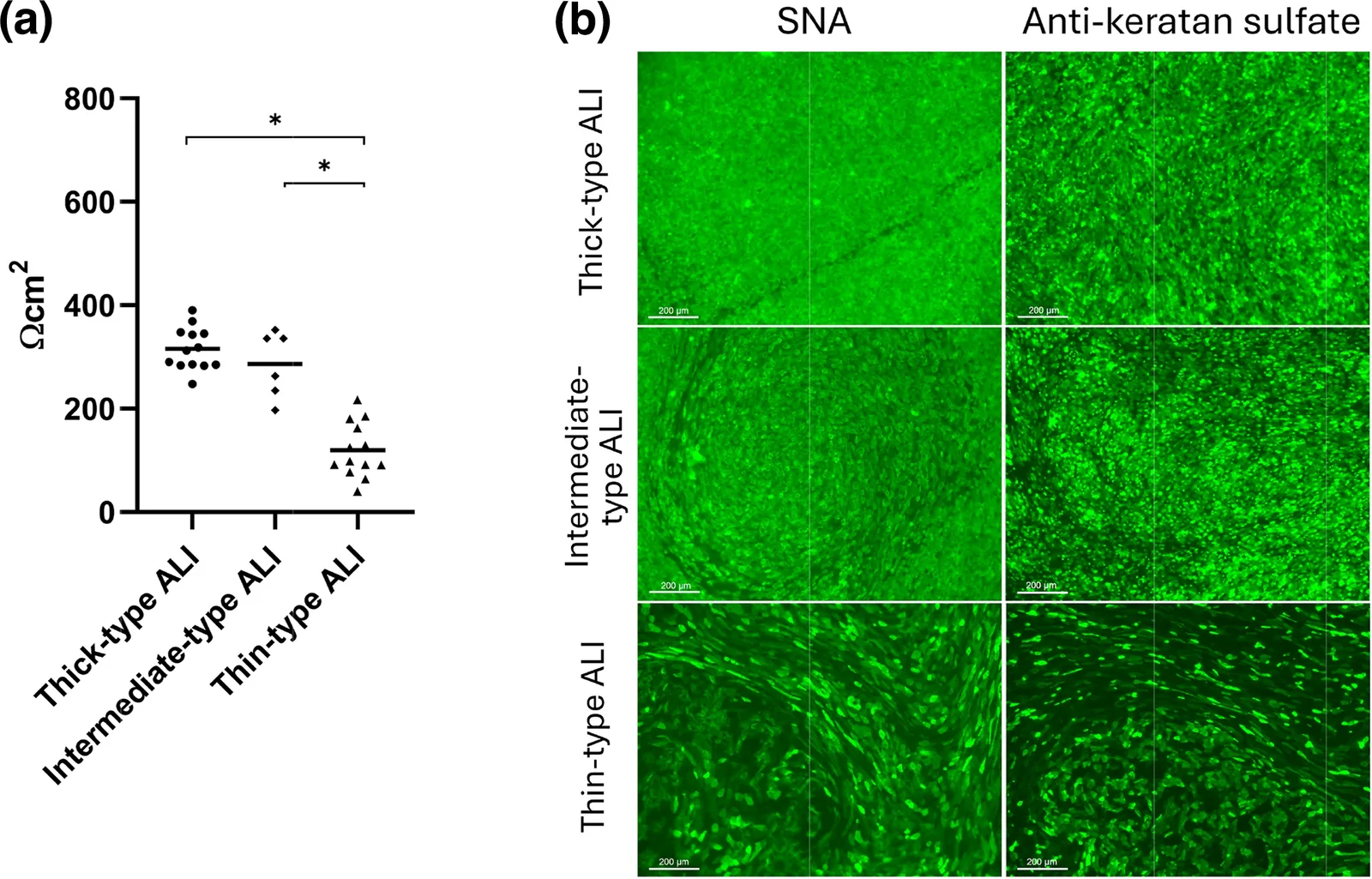
Epithelial barrier and apical surface integrity in the classified HBTEC-ALI culture system. (**a**) TEER was measured to evaluate the epithelial barrier function. Individual data points and the mean are shown. The number of samples for each group was as follows: Thick-type ALI (*n*=13), Intermediate-type ALI (*n*=6) and Thin-type ALI (*n*=13). Statistical analysis was performed using one-way ANOVA followed by Tukey’s multiple comparisons test (*P*<0.05). (**b**) The apical surfaces of HBTEC-ALI cultures were stained with FITC-conjugated *S. nigra* lectin (SNA), which detects *α*2,6-linked sialic acid, or with an anti-keratan sulphate antibody. Fluorescence signals were observed using a BZ-X810 fluorescence microscope. Scale bar, 200 µm.

### Impact of ALI culture types on respiratory viral replication

Based on previous results, we observed that the phenotype of the Thin-type ALI differed significantly from those of the Thick-type and Intermediate-type ALI. Owing to limitations in the number of wells and available resources, a separate comparison between the Thick-type and Intermediate-type ALI was not feasible. Therefore, the viral replication capacity was compared between the combined Thick- and Intermediate-type group and the Thin-type ALI. Most respiratory viruses tended to replicate more efficiently in Thick/Intermediate-type than in Thin-type ALI (Figs 4a and S3). In contrast, adenovirus replication was higher in Thin-type cultures, while bocavirus replication did not differ significantly (Figs 4a and S3). These findings suggest that the susceptibility of ALI cultures to viral replication varies depending on the virus.

**Fig. 4.**
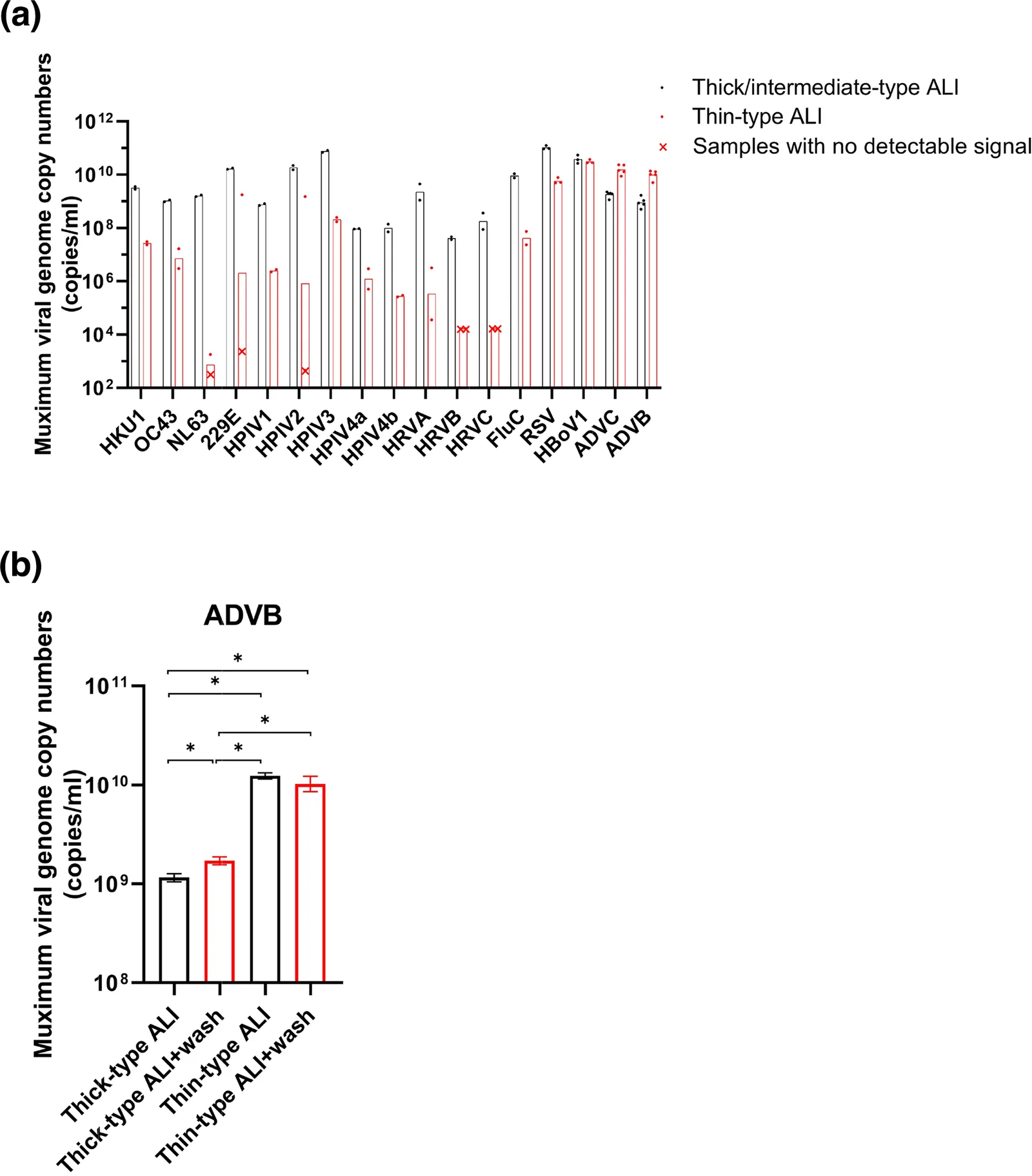
Comparison of viral replication. (**a**) Viral genome copy numbers in HBTEC-ALI cultures infected with various viruses were measured at 4, 7, 14 and 21 dpi, and the maximum viral genome copy number obtained from each individual culture during this observation period is shown. Because the timing of peak replication differed among viruses and individual cultures, the maximum value during the observation period was used for comparison. For each virus, the same viral genome copy number and inoculation volume were used across the different ALI culture types. Individual data points and the mean are indicated. Samples with no detectable signal (indicated by X) were assigned a Cp value of 40 (the assay detection limit) for mean calculation. This applied to NL63, 229E, HPIV2, HRVB and HRVC. The number of samples for each virus group was as follows: RSV and HBoV1 (*n*=3), ADVC and ADVB (*n*=5) and all other viruses (*n*=2). (**b**) ADVB genome copy numbers with or without apical wash fluid (wash) derived from Thick-type ALI were compared, and the maximum viral genome copy number for each individual culture during the observation period is shown. The same viral genome copy number and inoculation volume were used across the different ALI culture types. Data are presented as mean±sd. The number of samples for each group was as follows: Thick-type ALI with or without wash (*n*=3) and Thin-type ALI with or without wash (*n*=4). One-way ANOVA followed by Tukey’s multiple comparisons test was used (*P*<0.05). HKU1, OC43, NL63, 229E (HCoV-HKU1, OC43, NL63, 229E); HPIV1, 2, 3, 4a and 4b (human parainfluenza virus 1, 2, 3, 4a and 4b); HRVA, B, C (human rhinovirus A, B, C); FluC; RSV; HBoV1 (human bocavirus 1); ADVC (adenovirus species C); and ADVB (adenovirus species B).

To determine whether soluble apical factors inhibited adenovirus infection, viral inoculum was diluted with apical washes from Thick-type cultures and inoculated into both culture types. The apical wash fluid did not inhibit adenovirus replication of Thin-type ALI (Figs 4b and S3). These findings suggest that secreted components in the mucus layer do not significantly restrict infection under these conditions. However, it is still possible that cell-associated components, which are closely attached to the cell surface, play a role in modulating viral replication.

## Discussion

Among the differentiation markers examined, *KRT5* expression showed the clearest association with morphological phenotype and epithelial thickness. However, the present data do not establish a quantitative cutoff for classifying HBTEC-ALI cultures based on *KRT5* expression. Rather, *KRT5* expression appears to vary continuously with culture characteristics and may therefore serve as a practical complementary indicator of HBTEC-ALI culture status rather than as a standalone classification criterion. This observation suggests that basal cell-associated characteristics may be related to variation in epithelial structural maturation in this system, consistent with the important role of basal cells as tissue stem cells of the lung [26]. The differentiation process in ALI cultures begins sequentially with the appearance of basal cells, followed by the emergence of secretory cells and ciliated cells [27]. In this study, gene expression signatures characteristic of major airway epithelial cell types were detected even in Thin-type cultures, suggesting that differentiation toward these epithelial lineages had occurred to some extent. Differences among cultures were also associated with barrier-related properties, including TEER and apical glycan staining. These findings suggest that viral replication may be associated not only with cell type-associated gene expression but also with epithelial structural maturity and barrier integrity. Importantly, the relationships observed in this study should be interpreted as associations between morphological phenotypes and objectively measured characteristics rather than as evidence of objectively defined biological categories.

The reduced adenovirus replication observed in Thick/Intermediate-type ALI cultures may be explained in two possible ways. First, the adenovirus receptor coxsackievirus and adenovirus receptor (CAR) is predominantly localized beneath tight junctions [28]. ADVC primarily uses CAR for entry, whereas ADVB uses CD46 and desmoglein 2 (DSG2) as receptors [29–31]. Similar to CAR, DSG2 is localized to epithelial junctions [31, 32]. The stronger barrier integrity in Thick/Intermediate-type cultures, as indicated by higher TEER values, may restrict viral entry from the apical surface. These results are consistent with previous findings, indicating that adenovirus has higher infection efficiency when introduced from the basolateral side than from the apical side [32]. Furthermore, when adenovirus receptors are specifically expressed on the apical side of the cells, the efficiency of infection from that side increases [28].

Second, structural features of the apical surface may further limit viral access. In the airway epithelium, the mucus layer and the PCL are structurally separated, and the associated glycocalyx within the PCL prevents mucus penetration into the PCL and restricts access of adenovirus particles to the apical membrane [13, 14]. Keratan sulphate is present in both layers but is predominantly localized to the PCL, suggesting that its integrity is important for both layer separation and viral exclusion [14]. Reduced keratan sulphate staining in Thin-type cultures suggests compromised PCL integrity, potentially facilitating adenoviral access to the apical membrane. The lack of effect of apical wash fluid indicates that cell-associated structures rather than soluble mucus components predominantly influence viral accessibility under our conditions.

For most other respiratory viruses examined, replication tended to be higher in Thick/Intermediate-type cultures, potentially reflecting the greater number of cells accessible at the apical surface. This is consistent with the fact that many respiratory viruses use receptors localized on the apical surface [33–36]. In contrast, bocavirus replicated similarly across culture types. Its small particle size (25 nm) [37] may allow it to bypass physical barriers like the PCL more easily [13, 14]. While its receptor remains unidentified [38], bocavirus is thought to be able to access receptors on both the apical and basolateral surfaces [39], enabling efficient infection even in Thin-type cultures with fewer apical cells. These characteristics may explain the lack of significant differences in bocavirus replication.

This study has several limitations. First, the mechanisms underlying the emergence of different ALI morphologies remain unclear. Previous studies have reported that increased passage number can affect the morphological characteristics of primary airway epithelial cultures [8]. However, all cultures compared in the present study were generated using the same passage number, and Thick-, Intermediate- and Thin-type cultures were observed in all donor lots. Therefore, the morphological differences observed here are unlikely to be explained by donor or passage number alone. Second, the Intermediate-type category was introduced to reflect the continuous variation in HBTEC-ALI morphology, as some cultures exhibited intermediate features that could not be clearly assigned to either Thick- or Thin-type based on microscopic evaluation. Therefore, the viral replication analyses should be interpreted primarily as comparisons between Thick/Intermediate and Thin-type cultures, rather than as direct comparisons among three discrete morphological categories. Owing to resource and well number constraints, we were unable to directly compare Thick-type and Intermediate-type ALI cultures in viral replication assays. Consequently, these two categories were analysed as a combined group, which may have masked more subtle differences between them. Several virus-specific comparisons were based on only two independent cultures and should therefore be interpreted as exploratory trends rather than definitive differences. Although the same viral inoculum was applied to each insert, the number of differentiated epithelial cells at the time of infection was not directly quantified. Because epithelial thickness and tissue architecture differed among ALI culture types, the effective virus-to-cell ratio may have differed among cultures. Third, the classification of ALI cultures relied on visual morphological assessment by experienced investigators and therefore represents a practical morphological classification scheme rather than an entirely quantitative classification system. Future studies incorporating quantitative approaches, such as image analysis or objective functional measurements, may further improve classification reproducibility. Fourth, gene expression analyses were performed using bulk RNA. Therefore, the data indicate the presence of cell type-associated gene expression signatures but do not allow direct assessment of cellular composition, relative abundance or cellular heterogeneity. Fifth, while we identified associations between culture morphology, *KRT5* expression and viral replication, causal relationships were not directly established. Although antiviral responses such as cytokine production may also contribute to differences in viral replication, mechanistic analysis of these responses was beyond the scope of the present study.

In conclusion, readily observable morphological variation in HBTEC-ALI cultures was associated with differences in epithelial thickness, barrier-related properties and viral replication. Among the differentiation markers examined, *KRT5* showed the clearest association with culture morphology and epithelial thickness, suggesting its potential utility as an indicator of HBTEC-ALI culture status. Given the cost and limited availability of HBTEC-ALI cultures, assessment of culture status before experimental use may help optimize experimental design and interpretation. Our findings also indicate that no single morphological phenotype is necessarily optimal for all virological applications. Together with morphological assessment, *KRT5* expression may help characterize HBTEC-ALI cultures used for respiratory virus isolation and propagation.

## Supplementary material

10.1099/jgv.0.002361 Supplementary Material 1.

## Abbreviations

ADV: adenovirus
ALI: air–liquid interface
CAR: coxsackievirus and adenovirus receptor
CK5: cytokeratin 5
DMEM: Dulbecco’s modified Eagle’s medium
dpi: days post infection
DSG2: desmoglein 2
FluC: influenza C virus
HBoV1: human bocavirus 1
HBTEC-ALI: human bronchial/tracheal epithelial cell air–liquid interface
HCoV: human coronavirus
HPIV: human parainfluenza virus
HRV: human rhinovirus
PCL: periciliary layer
Real-time PCR: Real-time polymerase chain reaction
RSV: respiratory syncytial virus
RT-PCR: reverse transcription polymerase chain reaction
TEER: transepithelial electrical resistance
