# Supplementary material for: Optimizing respiratory virus replication in human bronchial/tracheal epithelial air–liquid interface cultures through practical quality indicators and key characteristics
Source: J Gen Virol. 2026 Nov 5;107(11):002361. doi: 10.1099/jgv.0.002361 (PMC13739291; doi:10.1099/jgv.0.002361)
Supplement: Supplementary file 1 [file jgv-107-02361-s001.pdf]

Supplemental figure. 1

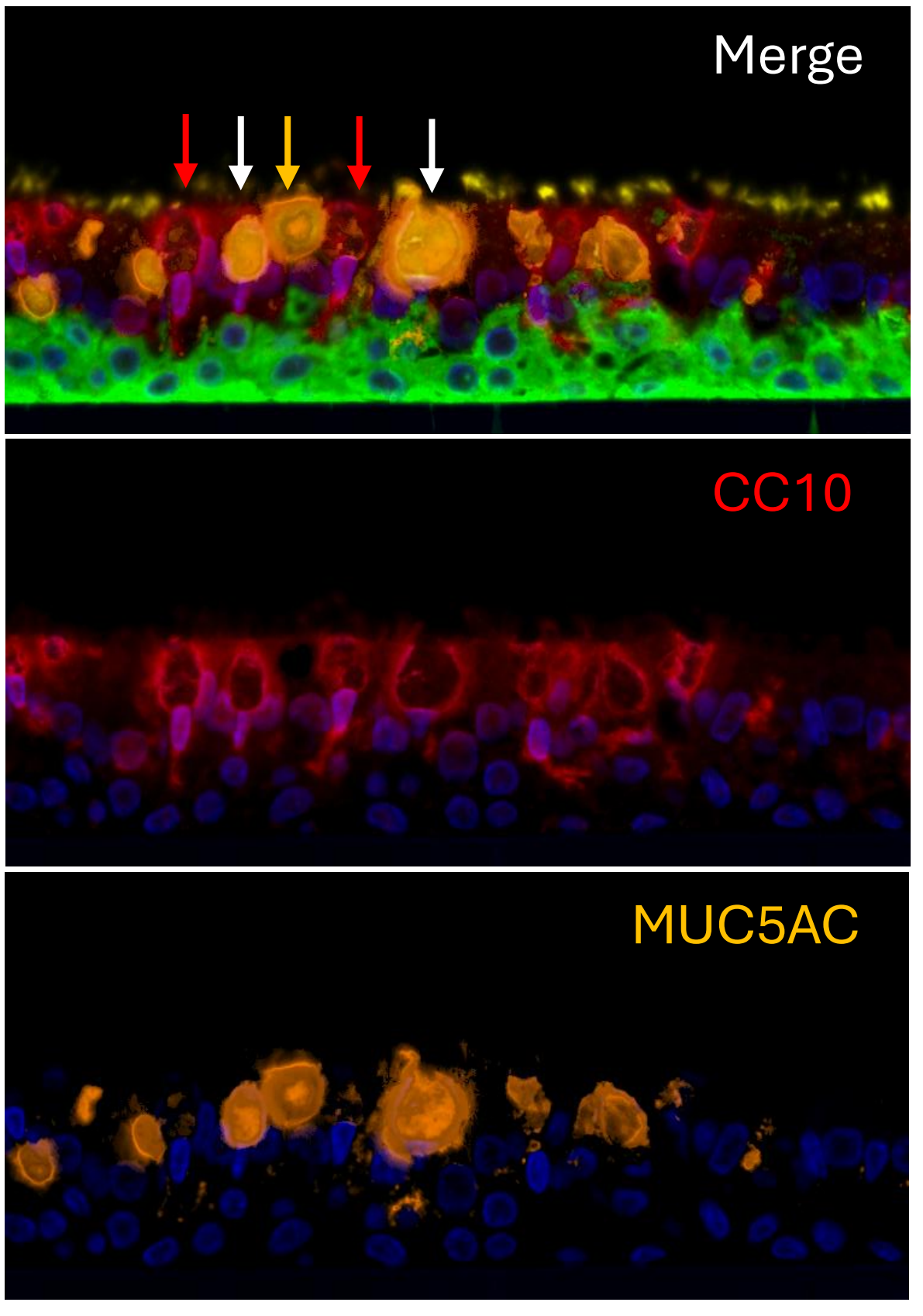

Fig. S1. Fluorescent quadruple staining of Thick-type ALI cultures for evaluation of cell types.
Differentiation markers were assessed: TUBA (acetylated alpha tubulin, yellow) for ciliated cells, CK5 (cytokeratin 5, green) for basal cells, CC10 (club-cell-specific 10 kDa protein, red) for secretory cells, and MUC5AC (mucin 5AC, orange) for goblet cells. Images were captured at ×200 magnification. White arrows indicate CC10/MUC5AC double-positive cells, red arrows indicate CC10 single-positive cells, and orange arrows indicate MUC5AC single-positive cells.

Supplemental figure. 2

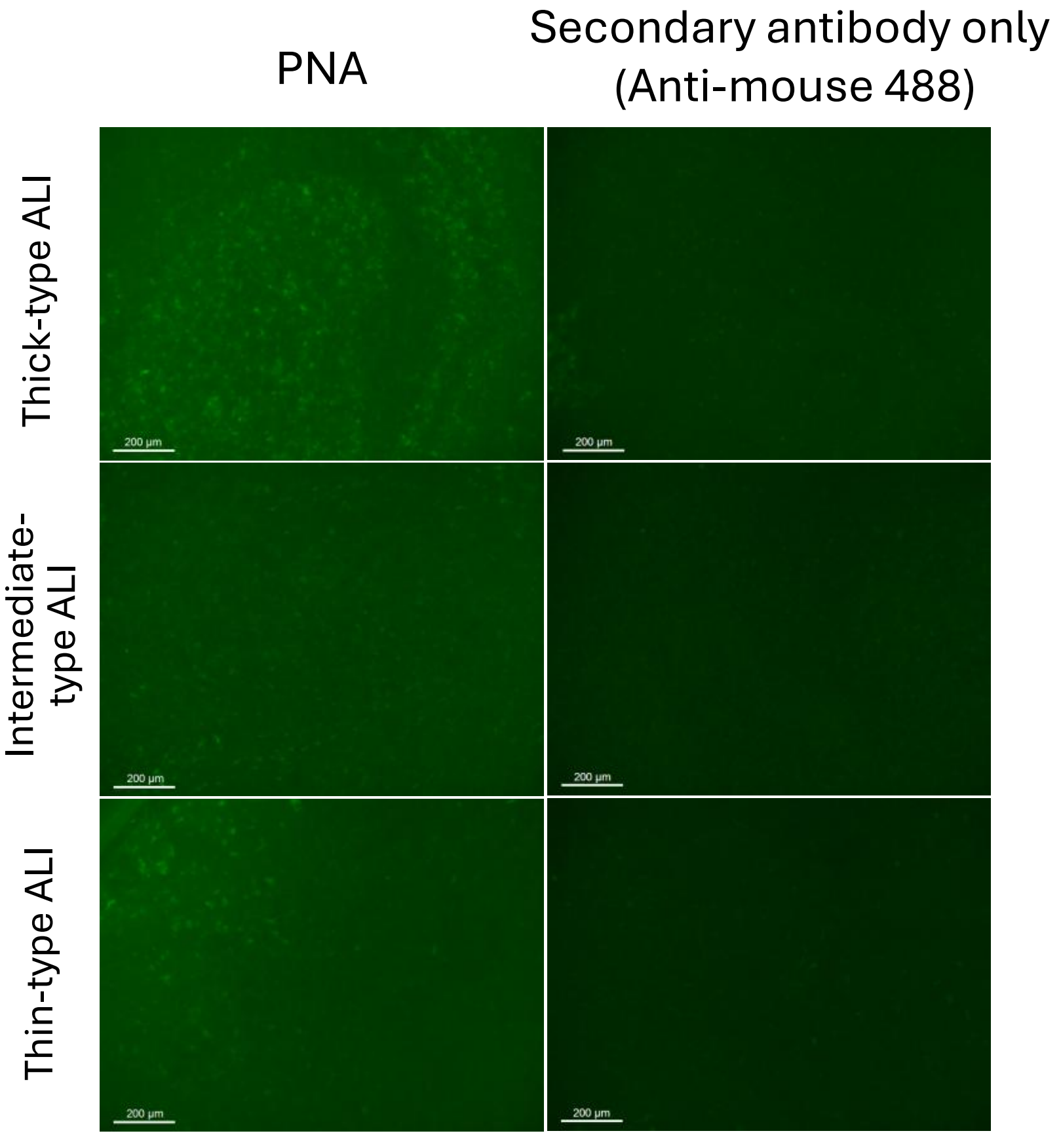

Fig. S2. Negative control staining of HBTEC-ALI cultures, corresponding to Fig. 3B.
For the negative controls, either PNA lectin was applied instead of SNA, or no primary antibody was used, confirming the specificity of the staining. Fluorescence was captured using a BZ-X810 fluorescence microscope. Scale bar, 200 µm.

Supplemental figure. 3

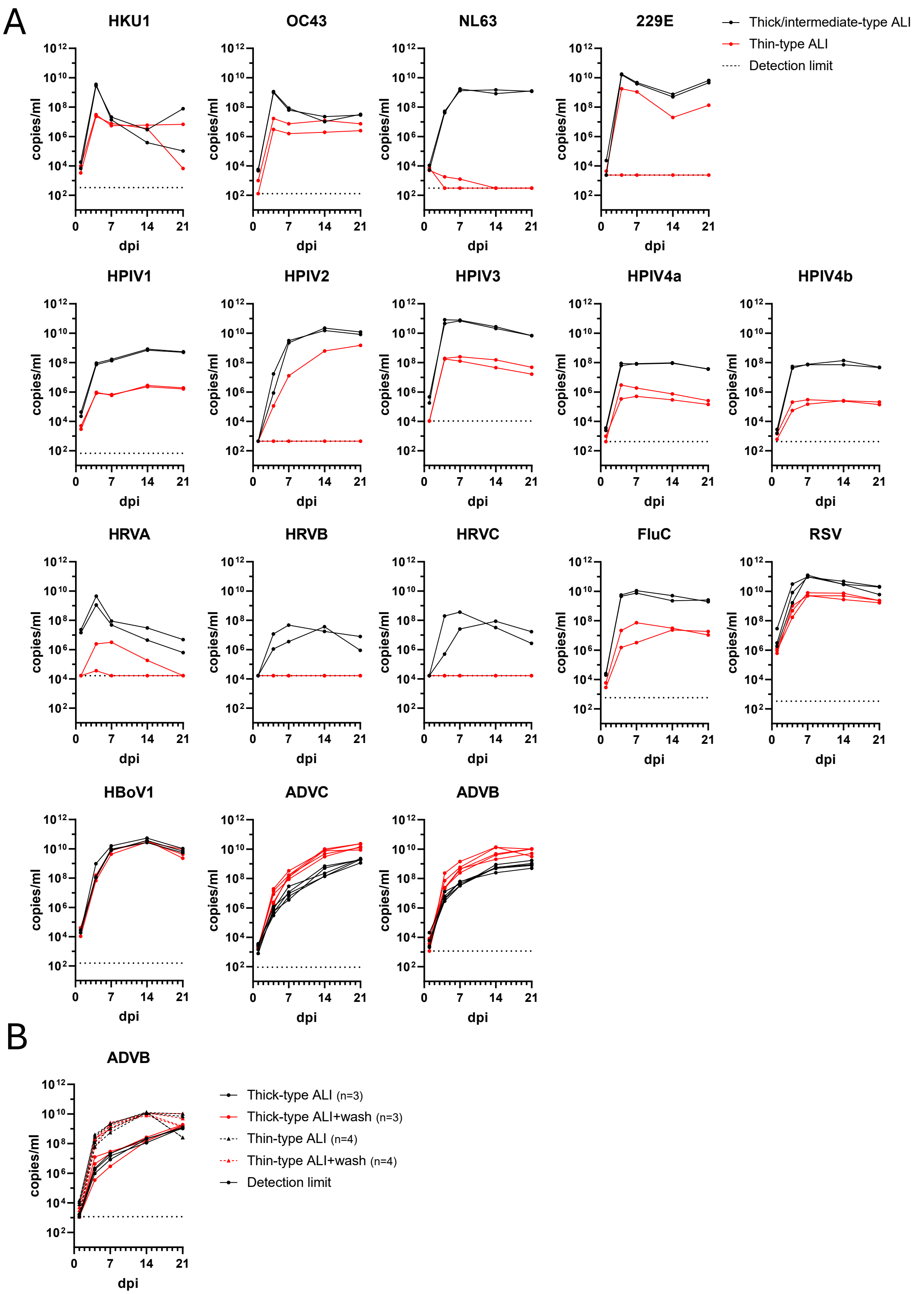

Fig. S3. Time course of viral genome replication.
(A) Viral genome copy numbers were measured over time in HBTEC-ALI cultures infected with various viruses. Samples with no detectable signal were assigned a Cp value of 40 (the assay detection limit). Sample sizes were as follows: RSV and HBoV1 (n = 3), ADVC and ADVB (n = 5), all other viruses (n = 2). These time-course data correspond to Fig. 4A. (B) ADVB genome copy numbers were measured over time in HBTEC-ALI cultures in the presence or absence of apical wash fluid (wash) derived from Thick-type ALI cultures. These time-course data correspond to Fig. 4B. Abbreviations: HKU1, OC43, NL63, 229E (human coronavirus-HKU1, OC43, NL63, 229E); HPIV1, 2, 3, 4a, 4b (human parainfluenza virus 1, 2, 3, 4a, 4b); HRVA, B, C (human rhinovirus A, B, C); FluC (influenza C virus); RSV (respiratory syncytial virus); HBoV1 (human bocavirus 1); ADVC (adenovirus species C); and ADVB (adenovirus species B).
